# Statistical image analysis of longitudinal RAVENS images

**DOI:** 10.3389/fnins.2015.00368

**Published:** 2015-10-20

**Authors:** Seonjoo Lee, Vadim Zipunnikov, Daniel S. Reich, Dzung L. Pham

**Affiliations:** ^1^Department of Psychiatry and Biostatistics, Columbia UniversityNew York, NY, USA; ^2^New York State Psychiatric InstituteNew York, NY, USA; ^3^Department of Biostatistics, Bloomberg School of Public Health, Johns Hopkins UniversityBaltimore, MD, USA; ^4^Division of Neuroimmunology and Neurovirology, National Institute of Neurological Disorders and Stroke, National Institutes of HealthBethesda, MD, USA; ^5^Center for Neuroscience and Regenerative Medicine, The Henry M. Jackson Foundation for the Advancement of Military MedicineBethesda, MD, USA

**Keywords:** longitudinal functional principal component analysis, regional analysis of volumes examined in normalized space, voxel-based morphometry, multiple sclerosis, brain volume measurement

## Abstract

Regional analysis of volumes examined in normalized space (RAVENS) are transformation images used in the study of brain morphometry. In this paper, RAVENS images are analyzed using a longitudinal variant of voxel-based morphometry (VBM) and longitudinal functional principal component analysis (LFPCA) for high-dimensional images. We demonstrate that the latter overcomes the limitations of standard longitudinal VBM analyses, which does not separate registration errors from other longitudinal changes and baseline patterns. This is especially important in contexts where longitudinal changes are only a small fraction of the overall observed variability, which is typical in normal aging and many chronic diseases. Our simulation study shows that LFPCA effectively separates registration error from baseline and longitudinal signals of interest by decomposing RAVENS images measured at multiple visits into three components: a subject-specific imaging random intercept that quantifies the cross-sectional variability, a subject-specific imaging slope that quantifies the irreversible changes over multiple visits, and a subject-visit specific imaging deviation. We describe strategies to identify baseline/longitudinal variation and registration errors combined with covariates of interest. Our analysis suggests that specific regional brain atrophy and ventricular enlargement are associated with multiple sclerosis (MS) disease progression.

## 1. Introduction

Magnetic resonance imaging (MRI) is commonly used in the study of brain structure. Many studies are based on measurements of tissue volumes within a number of predefined regions of interest (ROIs); for example, see Bartzokis et al. (2001) and Bermel et al. (2003). Although ROI analysis can directly quantify the volume of structures and reduce the dimensionality of images, the ROIs have to be defined before the analysis is conducted. In disease studies, this can be difficult without sufficient prior knowledge about what and how various regions will be affected. Moreover, ROI based measurements can be time-consuming and labor-intensive. The results of the analysis will depend on the quality of the ROI delineation and thus depend upon the experience of the operator and accuracy of segmentation algorithms.

**Figure 1:**
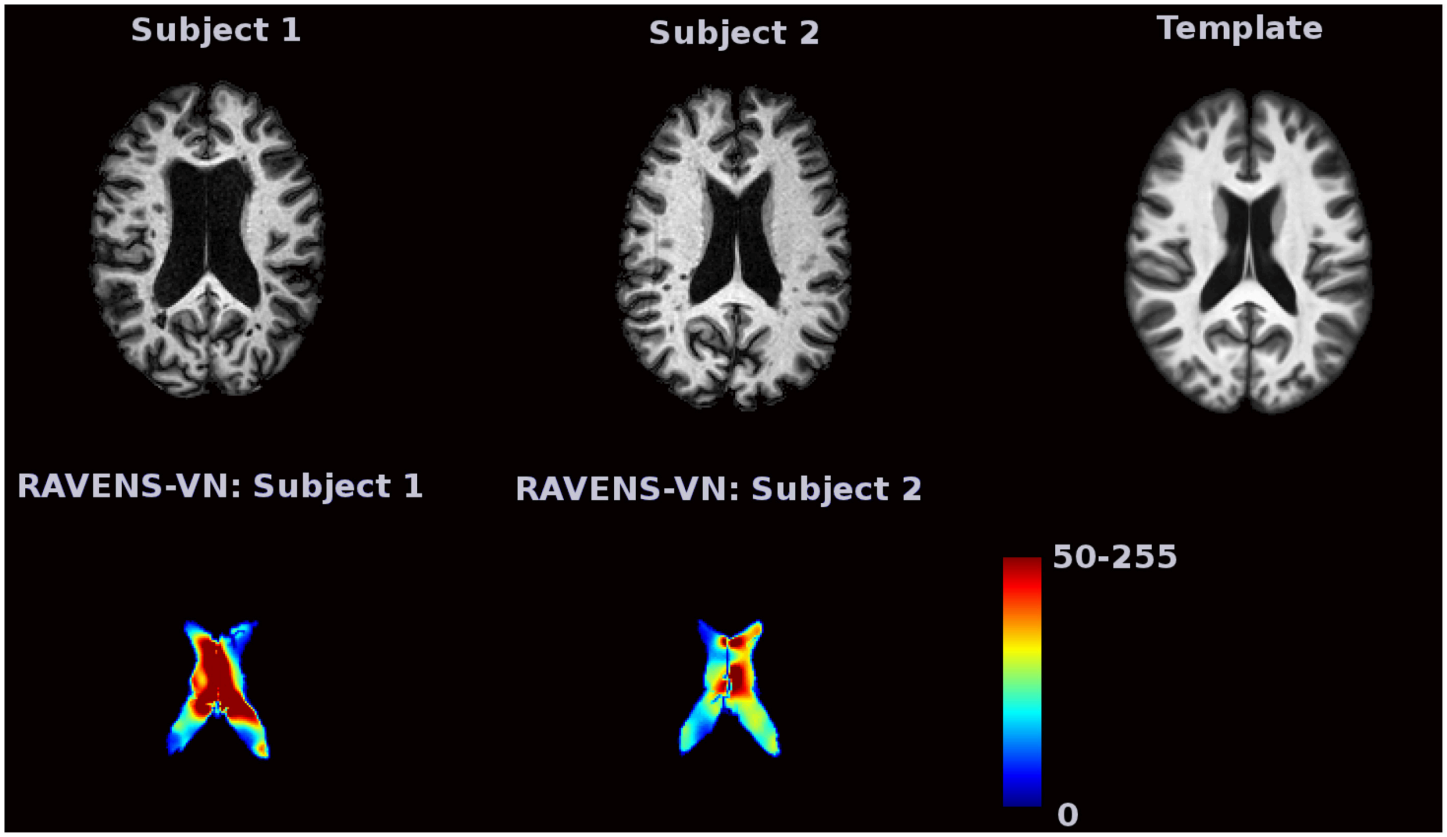
**The image intensities of the RAVENS image represent regional volumes relative to those of the template**. Red color represents high intensity and blue color represents lower intensity. The first brain, having a larger ventricle than the template brain, has brighter intensities in the RAVENS image. The second brain, which has smaller ventricles, has lower intensities in the associated RAVENS image.

Voxel-based morphometry (VBM) is a complementary technique that measures local brain volumes in a normalized space and thus does not suffer from these limitations (Ashburner and Friston, 2000, 2001). In this work, we consider Regional Analysis of Volumes Examined in Normalized Space (RAVENS), which registers each subject brain to a template of anatomy so that the intensities of the RAVENS image represent regional volumes relative to those of template (Shen and Davatzikos, 2002). In voxel-based morphometry methods such as RAVENS, segmentations of structures such as the ventricles, are mapped to a template brain. If a subject's ventricles are larger than the template brain's ventricles, each voxel in the ventricles need to be shrunken to be mapped to the template. This in turn increases the intensity of the RAVENS map at each voxel, implying a larger volume was present in the subject at each voxel. Figure 1 displays examples of ventricular RAVENS images in the template space. The first subject has much larger ventricles than the second subject (and template). Its RAVENS image of ventricles is displayed underneath the associated T1 image with red and blue colors representing higher and lower intensities, respectively. Subject 1 has larger ventricles, depicted by red in the RAVENS image. Similarly, the second brain, having a smaller ventricle than that of the first subject, has lower intensities in its RAVENS image, depicted by yellow and cyan in RAVENS image. By applying statistical VBM analysis of RAVENS images (RAVENS-VBM) to the resulting spatial distributions of gray matter (GM), white matter (WM), and ventricular cerebrospinal fluid (CSF), local atrophy or enlargement can be detected if the intensities significantly change across subjects.

In many disease studies, *longitudinal patterns* of brain structure between and within control and patient groups are of interest. Such studies are often based on ROI volume measurements followed by statistical analysis, such as a linear mixed model. Several neuroimaging software platforms, including: FSL (Smith et al., 2004), the SPM-VBM toolbox (available at http://dbm.neuro.uni-jena.de/vbm) and SurfStat (Worsley et al., 2009), support flexible longitudinal models. Statistical inference of the contrast between two different time points is the most commonly used approach (Bendfeldt et al., 2011). Numerous other approaches for longitudinal imaging data have been proposed for prediction. The methods include support vector machine classifiers (Chen and Bowman, 2011) and Bayesian spatial models (Derado et al., 2013).

In practice, there are frequently cases that VBM does not find significant longitudinal trend. Possible causes are (1) the chosen statistical method is not sophisticated enough to extract longitudinal information; (2) a substantial amount of visit-to-visit variation to longitudinal signals exists; (3) heterogeneous longitudinal patterns exist within the diseases population.

The obvious solution to overcome such limitations is to combine the VBM analysis with more sophisticated statistical methods such as linear mixed models. However, for the first two cases, hypothesis driven VBM analyses cannot further exploit the data. In that case, figuring out the underlying structures of variation in the longitudinal data would be of interest. Further, we want to quantify the longitudinal and cross-sectional variability, and the association between each subject and their spatial patterns.

Thus, our main goal is to introduce a new statistical framework for longitudinal VBM analysis. To achieve the goal, we consider a data-driven analysis to provide a more complete statistical framework to analyze high-dimensional longitudinal brain images. A framework to allow for this conceptual partition of variability is longitudinal functional principal component analysis (LFPCA; Greven et al., 2011). This method was originally proposed for low to moderate dimensional functional data and was extended to high dimensional data by Zipunnikov et al. (2014). The main idea of high-dimensional inference is based on projecting onto the intrinsic low dimensional space spanned by high-dimensional vectors (Di et al., 2009; Zipunnikov et al., 2011b). More precisely, we start by modeling the observed data with high-dimensional longitudinal functional principal component analysis (HD-LFPCA). Each RAVENS ventricular image is unfolded into a *p* × 1 dimensional vector, where *p* ≈ 80, 000 is the number of voxels in the RAVENS ventricular image. These vectors are decomposed in their baseline, longitudinal and visit-to-visit components; each component is then estimated from the data. The method takes only a few minutes on a standard PC.

In this paper we focus on LFPCA as a useful tool for longitudinal voxel-based analyses, particularly to quantify cross-sectional and longitudinal variability in the data. The simulation study illustrates the application of LFPCA to a simplified imaging setting. It demonstrates that LFPCA effectively separates longitudinal, cross sectional, and other variations. Notably, the simulation study shows that LFPCA can separate registration errors from baseline and longitudinal components of interest.

## 2. Materials and methods

### 2.1. Participants

Forty eight MS patients (aged 42±12 years at baseline) were enrolled in a longitudinal study of brain volume change. The study population included 33 female and 16 male patients; 28 patients with relapsing-remitting MS (RRMS), 13 patients with secondary progressive MS (SPMS), 5 patients with primary progressive MS (PPMS) and 2 patients with clinically isolated syndrome (CIS). One hundred forty eight T1 images have been acquired, with three images per subject for 44 subjects and 4 images per subject for 3 subjects. The average time interval between scans was 368 days (±27). All images were spatially normalized via registration of T1 maps into the mean template, generated using Advanced Normalization Tools (Avants et al., 2010, 2011) from 30 randomly chosen MS patients among those with more than three visits. Ethical approval for the study was granted by IRB-2 and Johns Hopkins Medicine Institutional Review Board. All participants signed their fully informed consent.

### 2.2. MRI protocol and image analysis

High resolution 3D magnetization-prepared rapid acquisition of gradient echoes (MPRAGE; acquired resolution: 1.1 × 1.1 × 1.1 mm; TR:~10 ms; TE: 6 ms; TI = 835 ms; flip angle: 8°; SENSE factor:2; averages:1) were acquired on a 3.0 T MRI scanner (Intera, Philips Medical Systems).

In the processing, the follow-up images are affinely registered to their baselines via FMRIB's Linear Image Registration Tool (Jenkinson et al., 2002). All T1 images were segmented into GM, WM, VN, and lesions with Lesion-TOADS (Shiee et al., 2010) that was specifically designed for tissue and MS lesion segmentation. In general, as MS progresses, multifocal lesions in the white matter develop, and newly developed legions can cause inaccuracies in the registration and RAVENS map computation. Thus, we masked those lesions in the registration using the Lesions-TOADS software. After segmentation, the final tissue maps of GM, WM, and VN were normalized using HAMMER-SUITE (Shen and Davatzikos, 2002) to generate RAVENS images. Finally, the RAVENS maps were separately smoothed with 4 mm FWHM using SPM8.

### 2.3. Longitudinal functional principal component analysis

In this section, we provide a description of the original LFPCA approach developed by Greven et al. (2011) and its extension for high-dimensional data analysis (Zipunnikov et al., 2014). Throughout this section, we refer to both as LFPCA.

#### 2.3.1. Random intercept and random slope model

Consider a longitudinal brain imaging study with subjects labeled by index *i* with each visit indexed by *j* and scan time by variable *t*_*ij*_ for *j* = 1, …, *J*_*i*_. Each image is unfolded into a *p*-dimensional column vector **y**_*ij*_(*v*); the index *v* of each entry corresponds to a particular location in the brain for each subject and visit in normalized space. A random slope and random intercept model is commonly used to analyze longitudinal data, and it has been extended to functional (Greven et al., 2011) and imaging (Zipunnikov et al., 2014) studies as follows:

(2.1) $$y_{ij}(v)=\eta (v,t_{ij})+x_{i,0}(v)+x_{i,1}(v)t_{ij}+W_{ij}(v),$$

where *y*_*ij*_(*v*) denotes the image intensity at voxel *v*, η(*v, t*_*ij*_) is a fixed main effect, and *x*_*i*, 0_(*v*) and *x*_*i*, 1_(*v*) denote the random intercept and random slope for subject *i*, respectively. The term *W*_*ij*_(*v*) is a random subject-visit specific imaging deviation, which is assumed to be a zero mean, second-order stationary random process uncorrelated with ${\text{X}}_{i}\left(v\right)={\left(x_{i,0}\left(v\right),x_{i,1}\left(v\right)\right)}^{⊤}$. The covariance operators of **X**_*i*_(*v*) and *W*_*ij*_(*v*) are denoted as ${\text{K}}^{X}\left(v_{1},v_{2}\right)$ and ${\text{K}}^{W}\left(v_{1},v_{2}\right)$, respectively.

While this is a natural and relatively simple model for longitudinally observed data, the scale of the problem requires aggressive dimensionality reduction. LFPCA reduces dimensionality by projecting onto the subspaces which explain principal directions of variation in the data. In model (2.1), there are two sources of variation: subject-to-subject, captured by **X**_*i*_, and visit-to-visit within a subject, captured by **W**_*ij*_ and the model assumption on **X**_*i*_ and **W**_*ij*_ in (2.1) allows us to partition the variation of the data and LFPCA models latent processes **X**_*i*_ and **W**_*ij*_ using a Karhunen-Loeve (K-L) expansion (Karhunen, 1947; Loève, 1948).

The K-L expansion decomposes the two latent processes as ${\text{X}}_{i}\left(v\right)=\overset{\infty }{\underset{k=1}{\sum }}\xi_{ik}\phi_{k}^{X}\left(v\right)$ and $W_{ij}\left(v\right)=\overset{\infty }{\underset{l=1}{\sum }}\zeta_{ijl}\phi_{l}^{W}\left(v\right)$, where $\phi_{k}^{X}=\left(\phi_{k}^{X,0},\phi_{k}^{X,1}\right)$ and $\phi_{l}^{W}$ are the eigenfunctions of ${\text{K}}^{X}\left(v_{1},v_{2}\right)$ and ${\text{K}}^{W}\left(v_{1},v_{2}\right)$, respectively, such that

$$\begin{array}{l} K^{X}(v_{1},v_{2})=\left(\begin{array}{cc} K_{00}^{X}(v_{1},v_{2}) & K_{10}^{X}(v_{1},v_{2})\\ K_{01}^{X}(v_{1},v_{2}) & K_{11}^{X}(v_{1},v_{2}) \end{array}\right)\\ \;=\sum_{k=1}^{N_{X}}\lambda_{k}^{X}\phi_{k}^{X}(v_{1}){\left\{\phi_{k}^{X}(v_{2})\right\}}^{⊤}. \end{array}$$

LFPCA truncates K-L representations and represents observed data through a linear mixed-effects model:

(2.2) $$\begin{array}{l} y_{ij}(v)=\eta (v,t_{ij})+\sum_{k=1}^{N_{X}}\xi_{ik}\phi_{k}^{X,0}(v)+t_{ij}\sum_{k=1}^{N_{X}}\xi_{ik}\phi_{k}^{X,1}(v)\\ \;+\sum_{l=1}^{N_{W}}\zeta_{ijl}\phi_{l}^{W}(v),\\ (\xi_{ik_{1}},\xi_{ik_{2}})\sim (0,0,\lambda_{k_{1}}^{X},\lambda_{k_{2}}^{X},0);(\zeta_{il_{1}},\zeta_{il_{2}})\sim (0,0,\lambda_{l_{1}}^{W},\lambda_{l_{2}}^{W},0), \end{array}$$

where “$\cdot \sim \left(\mu_{1},\mu_{2};\sigma_{1}^{2};\sigma_{2}^{2};\rho \right)$” denotes that a pair of variables has a distribution with mean (μ_1_, μ_2_), variance $\sigma_{1}^{2},\sigma_{2}^{2}$, and correlation ρ. We assume that λ_*k*_1__ ≥ λ_*k*_2__ if *k*_1_ ≤ *k*_2_. Since **X**_*i*_(*v*) and *W*_*ij*_(*v*) are uncorrelated, the scores ${\left\{\xi_{ik}\right\}}_{k=1}^{\infty }$ and ${\left\{\zeta_{ijl}\right\}}_{l=1}^{\infty }$ are also uncorrelated. A very important characteristic of model (2.2) is that both *N*_*X*_ and *N*_*W*_ are expected to be small in most applications.

For the unfolded vector, (2.2) can be rewritten as ${\text{y}}_{ij}=\eta \left(t_{ij}\right)+\Phi_{X}^{0}\xi_{i}+t_{ij}\Phi_{X}^{1}\xi_{i}+\Phi_{W}\zeta_{ij}$, where ${\text{y}}_{ij}={\left(y_{ij}\left(v_{1}\right),\ldots ,y_{ij}\left(v_{p}\right)\right)}^{⊤}$ is a *p* × 1 dimensional vector; $\phi_{k}^{X,0}$,$\phi_{k}^{X,1}$, and $\phi_{l}^{W}$ are *p* × 1 eigenvectors; $\Phi_{X}^{s}=\left(\phi_{1}^{X,s},\ldots ,\phi_{N_{X}}^{X,s}\right)$ for *s* = 0, 1; $\Phi_{W}=\left(\phi_{1}^{W},\ldots ,\phi_{N_{W}}^{W}\right)$; $\xi_{i}={\left(\xi_{i1},\ldots ,\xi_{iN_{X}}\right)}^{⊤}$;$\zeta_{i}={\left(\zeta_{i1},\ldots ,\zeta_{iN_{W}}\right)}^{⊤}$.

In brain imaging data analysis, LFPCA can separate biological signals from non-biological artifacts. For example, registration errors due to structural differences between subjects can be captured by baseline subject-specific components $\Phi_{X}^{0}$ and scanner variability can be captured by visit-to-visit components **Φ**_*W*_. This will be illustrated via an extensive simulation experiment in Section 3.1.

The fixed effect η(*v, t*_*ij*_) can be estimated in a number of ways (Greven et al., 2011). The analyses in the later sections simply use the sample mean across all the image observations. Once η(*v, t*_*ij*_) is estimated by the sample mean $\tilde{\eta }\left(v,t_{ij}\right)$, the longitudinal eigenanalysis is applied to the residual images ${ỹ}_{ij}\left(v\right)=y_{ij}\left(v\right)-\tilde{\eta }\left(v,t_{ij}\right)$ that are modeled as follows:

(2.3) $${\tilde{y}}_{ij}=\Phi_{X}^{0}\xi_{i}+t_{ij}\Phi_{X}^{1}\xi_{i}+\Phi_{W}\zeta_{ij}.$$

#### 2.3.2. LFPCA estimation

Zipunnikov et al. (2014) modified the original approach of Greven et al. (2011) and developed a method of moments estimator based on quadratics of ${\tilde{\text{y}}}_{ij}$. The *p* × *p*-covariance of ${\tilde{\text{y}}}_{ij_{1}}$ and ${\tilde{\text{y}}}_{ij_{2}}$ is given by

(2.4) $$\begin{array}{l} \text{E}\left\{{\tilde{y}}_{ij_{1}}{\tilde{y}}_{ij_{2}}^{⊤}\right\}=K_{00}^{X}+t_{ij_{1}}K_{10}^{X}+t_{ij_{2}}K_{X}^{10}+t_{ij_{1}}t_{ij_{2}}K_{X}^{11}+\delta_{j_{1},j_{2}}K^{W},\\ \;j_{1},j_{2}=1,\ldots ,J_{i}, \end{array}$$

where δ_*i, j*_ = 1 if *i* = *j* and δ_*i, j*_ = 0 otherwise. Model (2.4) can be rewritten in terms of unfolded vectors ${\text{K}}^{v}=\left\{\text{vec}{\text{K}}_{00},\text{vec}{\text{K}}_{01},\text{vec}{\text{K}}_{10},\text{vec}{\text{K}}_{11},\text{vec}{\text{K}}^{W}\right\}$ and **f**_*i*_*j*__1_*j*_2__ $={\left(1,t_{ij_{2}},t_{ij_{1}},t_{ij_{1}}t_{ij2},\delta_{j_{1},j_{2}}\right)}^{⊤}$ such that $E\text{vec}{\tilde{\text{y}}}_{ij_{1}}{\tilde{\text{y}}}_{ij_{2}}^{⊤}={\text{K}}^{v}{\text{f}}_{ij_{1}j_{2}}$. By concatenating all vectors across all subjects and visits we obtain a moment matrix identity for the *p*^2^ × *m* matrix **Y**: E**Y** =**K**^*v*^**F**, where $m=\overset{N}{\underset{i=1}{\sum }}J_{i}^{2}$. Then covariance parameters **K**^*v*^ can be unbiasedly estimated by using ordinary least squares (OLS): ${\hat{\text{K}}}^{v}=\text{Y}{\text{F}}^{⊤}{\left({\text{FF}}^{⊤}\right)}^{-1}$.

The covariance operators **K**^*X*^ and **K**^*W*^ are 2*p* × 2*p* and *p* × *p* dimensional, respectively. For high-dimensional functional data, storing or diagonalizing these matrices is not feasible. Zipunnikov et al. (2014) proposed HD-LFPCA, a novel estimation approach that takes advantage of an intrinsically small dimension of the space spanned by high-dimensional data vectors. First we form a *p* × *J*_*i*_ dimensional matrix ${\tilde{\text{y}}}_{i}$, where column *j* corresponds to a demeaned-RAVENS image obtained for subject *i* at visit *j*. The *p* × *J* dimensional data matrix $\tilde{\text{y}}=\left({\tilde{\text{y}}}_{1};\ldots ;{\tilde{\text{y}}}_{n}\right)$ is formed by column-binding the blocks of data corresponding to each subject, where $J=\overset{N}{\underset{i=1}{\sum }}J_{i}$. The data matrix can be decomposed as $\tilde{\text{y}}={\text{VSU}}^{⊤}$ using a singular value decomposition (SVD) approach. In the RAVENS image application, *J* = 148. Equation (2.3) can be rewritten as

(2.5) $${\tilde{y}}_{ij}=VSU_{ij}=\Phi_{X}^{0}\xi_{i}+t_{ij}\Phi_{X}^{1}\xi_{i}+\Phi_{W}\zeta_{ij}.$$

By multiplying with **V**^⊤^ to the left, we have

(2.6) $$\begin{array}{l} SU_{ij}=V^{⊤}\Phi_{X}^{0}\xi_{i}+t_{ij}V^{⊤}\Phi_{X}^{1}\xi_{i}+V^{⊤}\Phi_{W}\zeta_{ij}\\ \;=A_{X}^{0}\xi_{i}+A_{X}^{1}\xi_{i}+A_{W}\zeta_{ij}. \end{array}$$

We estimate ${\hat{\text{A}}}_{X}^{0},{\hat{\text{A}}}_{X}^{1}$, and ${\hat{\text{A}}}_{W}$ as described earlier, and estimate ${\hat{\Phi }}_{X}^{0}=\text{V}{\hat{\text{A}}}_{X}^{0}$, ${\hat{\Phi }}_{X}^{1}=\text{V}{\hat{\text{A}}}_{X}^{1}$, and ${\hat{\Phi }}_{W}=\text{V}{\hat{\text{A}}}_{W}$. Note that multiplying by **V**^⊤^ in Equation (2.5) reduces the model to its low-dimensional form (2.6), without losing the original correlation structure of the data. Once inference is conducted in model (2.6), then quantities of interest from model (2.5) can be estimated by pre-multiplying Equation (2.6) by **V**.

Principal scores ξ_*i*_ and ζ_*ij*_ are estimated via Best Linear Unbiased Predictions (BLUPs) as follows. The stacked vector of *i*th subject data, $\text{vec}{\tilde{\text{y}}}_{i}={\left({\tilde{\text{y}}}_{i1}^{⊤},\ldots ,{\tilde{\text{y}}}_{iJ_{J_{i}}}^{⊤}\right)}^{⊤}$, can be rewritten as $\text{vec}{\tilde{\text{y}}}_{i}={\text{B}}_{i}\omega_{i}$, where ${\text{B}}_{i}=\left({\text{B}}_{i}^{X};{\text{B}}_{i}^{W}\right)$, ${\text{B}}_{i}^{X}={\text{1}}_{J_{i}}⊗\Phi_{X}^{0}+{\text{T}}_{i}⊗\Phi_{X}^{1}$, ${\text{B}}_{i}^{W}={\text{I}}_{J_{i}}⊗\Phi_{W}$, where ${\text{T}}_{i}={\left(t_{i1},\ldots ,t_{iJ_{i}}\right)}^{⊤}$, $\omega_{i}={\left(\xi_{i}^{⊤},\zeta_{i}^{⊤}\right)}^{⊤}$, the subject level principal scores $\zeta_{i}={\left(\zeta_{i1}^{⊤},\ldots ,\zeta_{iJ_{i}}^{⊤}\right)}^{⊤}$, and **1**__*J*__*i*__ is a *J*_*i*_ × 1 vector of ones. Then the scores can be estimated as ${\hat{\omega }}_{i}={\left({\hat{\text{B}}}_{i}^{⊤}{\hat{\text{B}}}_{i}\right)}^{-1}{\hat{\text{B}}}_{i}^{⊤}\text{vec}{\tilde{\text{y}}}_{i}$. Due to linearity the estimated scores are the same in both models (2.5) and (2.6). Details of the matrix calculation and additional theoretical results of HD-LFPCA can be found in Zipunnikov et al. (2014).

The computed subject-specific principal component scores ξ_*i*_ are the derived composite scores computed for each linear trajectories based on the eigenvectors for subject-specific PCs. These scores can be used as predictors or outcomes in subsequent regression analyses to evaluate relationships between high-dimensional longitudinal trajectories and other variables of interest. Also, we can apply cluster analysis on the scores to uncover latent structure in the sample.

### 2.4. Classical VBM analysis using linear mixed model

First, we applied traditional VBM analysis using a linear mixed model to find a longitudinal trend. Many previous longitudinal studies have applied pairwise comparisons between two time points (Driemeyer et al., 2008). This study attempts to discover constant longitudinal trends over the time, i.e., focusing on the atrophy or enlargement rates. This may elucidate disease progression patterns of the patients. For the *i*th subject *j*th visit, the RAVENS image at voxel *v* follows the model:

(2.7) $$\begin{array}{l} y_{ij}(v)=\beta^{0}(v)+\beta^{1}(v)t_{ij}+b_{i}^{0}+b_{i}^{1}t_{ij}+\epsilon_{ij}(v),\\ \;b_{i}^{0}\sim N(0,\sigma_{0}^{2}(v)),\;b_{i}^{1}(v)\sim N(0,\sigma_{1}^{2}(v)),\\ \text{Cov}(b_{i}^{0}(v),b_{i}^{1}(v))=\sigma_{12},\;\epsilon_{ij}(v)\sim N(0,\sigma_{\epsilon }^{2}(v)), \end{array}$$

where β^0^(*v*) and β^1^(*v*) are the fixed-effect coefficients, $b_{i}^{0}$ and $b_{i}^{1}\left(v\right)$ are the random-effect coefficients for subject *i*, ϵ_*ij*_(*v*) is the error. The parameters are estimated based on maximum likelihood estimation and the *p*-values of the fixed effect parameters are compuated controlling for false discovery rate using (Benjamini and Yekutieli, 2001). We perform the statistical analysis in R (version 2.15.1).

## 3. Results

### 3.1. Simulated images

In this section, we present a simulation study to test the performance of LFPCA in RAVENS-VBM analysis. We investigate if LFPCA can identify subject-specific signals from noise, particularly registration errors, which often dominate signals in VBM analyses. Also, we identify cross-sectional and longitudinal variation when they exist.

**Figure 2:**
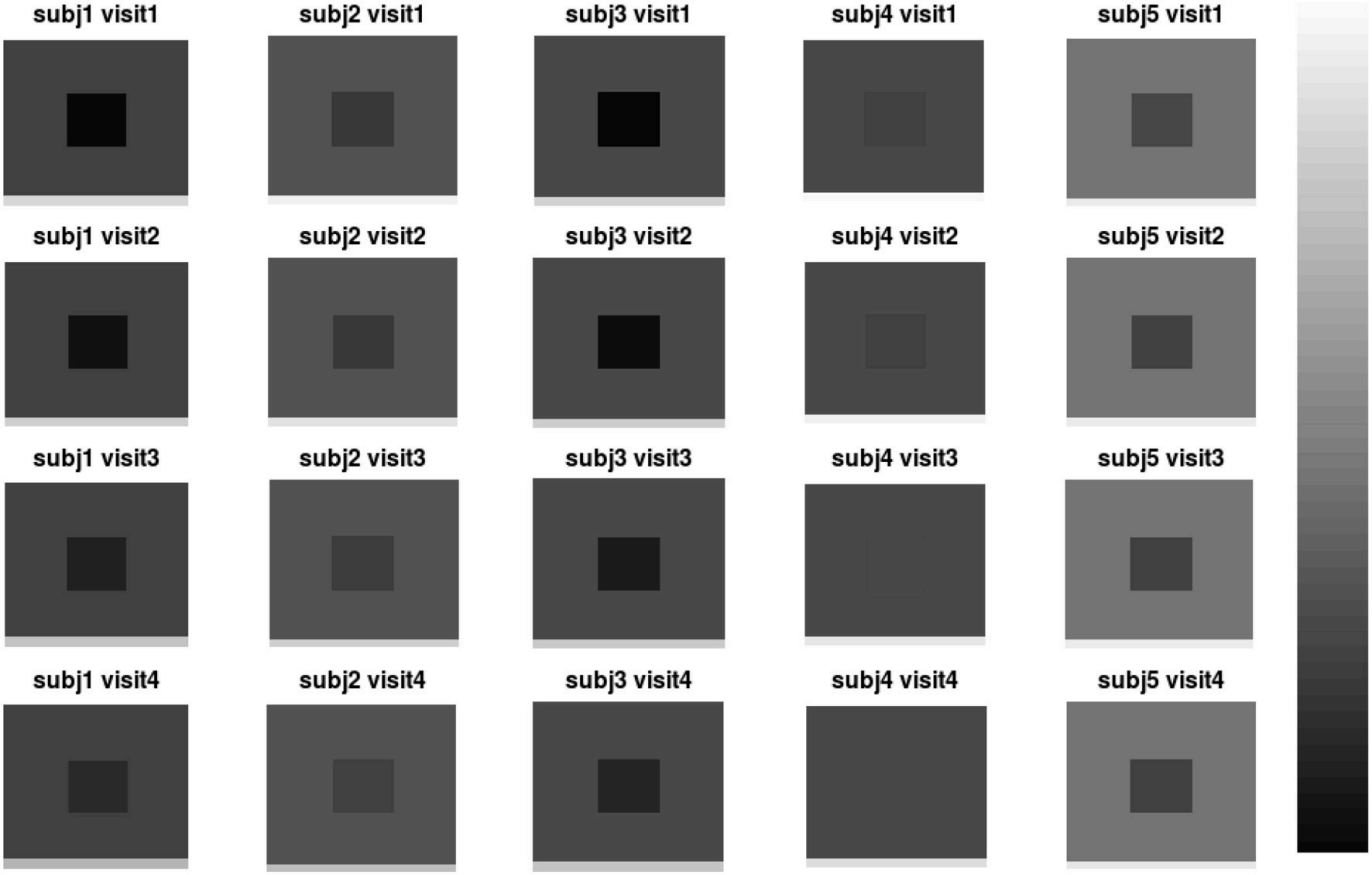
**Simulated data**. The longitudinal images consisting of four time points from five subjects are displayed.

We design a simulation study to mimic longitudinal analysis of RAVENS images. For the purpose of illustration, we use 2D images with 200 × 200 = 40, 000 pixels. We generate images from 50 subjects (*N* = 50) with three follow-ups. To replicate RAVENS processing routine, we assume that all images are registered to a template space. Figure 2 displays simulated RAVENS images from 5 randomly chosen subjects. Each column represents four longitudinally collected images of the same subject.

Each image mimics four canonical brain structures: background (B), white matter (W), ventricles (V), and gray matter (G). Those four components are simplified and shown as a background, a big square, a small square inside the big square, and a rectangle at the bottom, respectively. Registration errors are introduced via random rigid shifts of simulated structures as described below.

In Figure 2, the images from the first subject, which are displayed at the first column, show the longitudinal patterns. In the images, the color of V changes from darker gray to brighter gray, which represents longitudinal enlargements of V. Similarly, the colors of W and G changed to darker colors, which represent longitudinal atrophy.

**Figure 3:**
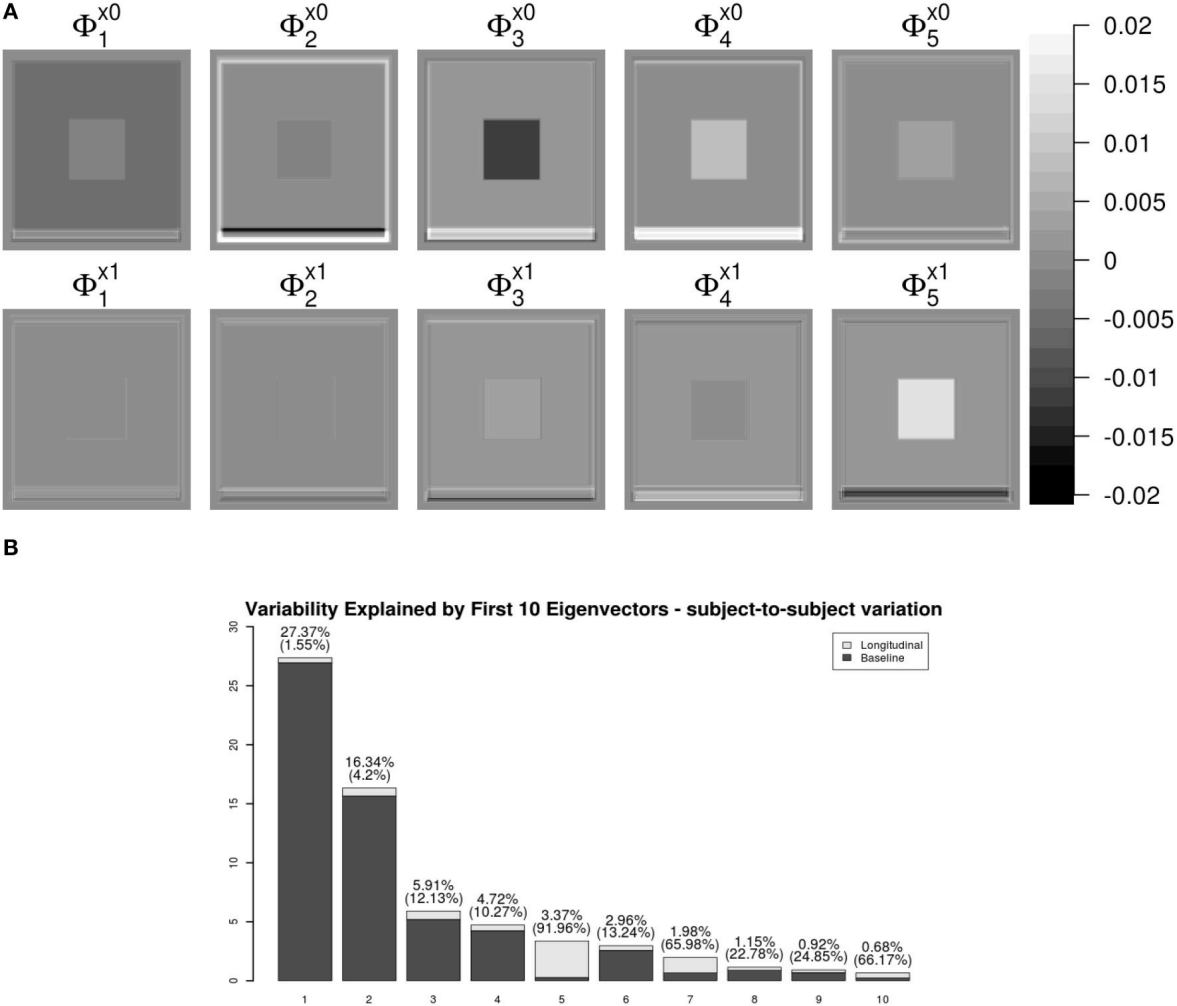
**LFPCA subject-specific components Φ_***X***_**. **(A)** Baseline ($\Phi_{X}^{0}$) and Longitudinal ($\Phi_{X}^{1}$) Components. **(B)** Variation explained by subject-specific components with ratios of longitudinal and baseline components.

Figure 3 shows the first five pairs of subject-specific components (**Φ**_*X*_). The baseline components ($\Phi_{X}^{0}$) are displayed in the top row and their corresponding longitudinal components ($\Phi_{X}^{1}$) are displayed in the second row. Each image is colored with a black(negative)-gray(0)-white(positive) color scheme. The first subject-specific component (Figure 3A first column) represents cross-sectional variations of the intensities of the W. The second component captures subject-specific registration errors, which only depend on cross-sectional variation. The third and fourth components represent the size of V and G. The fifth component shows longitudinal patterns of V and G. For a subject with positive score, the area V enlarges over the time and that of G shrinks, matching the truth used in simulation.

One useful feature of LFPCA is that contributions of the longitudinal and baseline components within each subject-specific component can be quantified on a [0, 1] scale. A subject-specific eigenvector is the stacked vector of baseline and longitudinal components: $\Phi_{X,k}={\left\{{\Phi_{X,k}^{0}}^{⊤},{\Phi_{X,k}^{1}}^{⊤}\right\}}^{⊤}$, such that $∥\Phi_{X,k}{∥}^{2}=∥\Phi_{X,k}^{0}{∥}^{2}+∥\Phi_{X,k}^{1}{∥}^{2}=1$. For each component, the variation or the contribution of the longitudinal component can be calculated as $\frac{∥\Phi_{X,j}^{1}{∥}^{2}}{∥\Phi_{X,k}^{0}{∥}^{2}+∥\Phi_{X,k}^{1}{∥}^{2}}$. Combined with the contribution of each subject-specific component to the total variation, Figure 3B displays variations explained by the first 10 subject-specific components with the proportion of the longitudinal components within each subject. Each bar plot intensity represents the amount of variation explained by each subject-specific component and is comprised of variations explained by the longitudinal component (dark) and the baseline component (bright). The top of each bar displays numerical values of the variation explained by the subject-specific component with the variation explained by the longitudinal component within the subject-specific component in parenthesis. Note that the fifth principal component has the highest longitudinal-baseline ratio among all 10 components. This provides a strong indication that the fifth component should be essentially treated as a longitudinal component. Using both visual and quantitative methods, we can conclude that the first four components represent baseline variation and registration error and the fifth component reveals longitudinal variation. In the data set, the longitudinal variation and baseline variations are independent, which agrees with the simulation setting.

**Figure 4:**
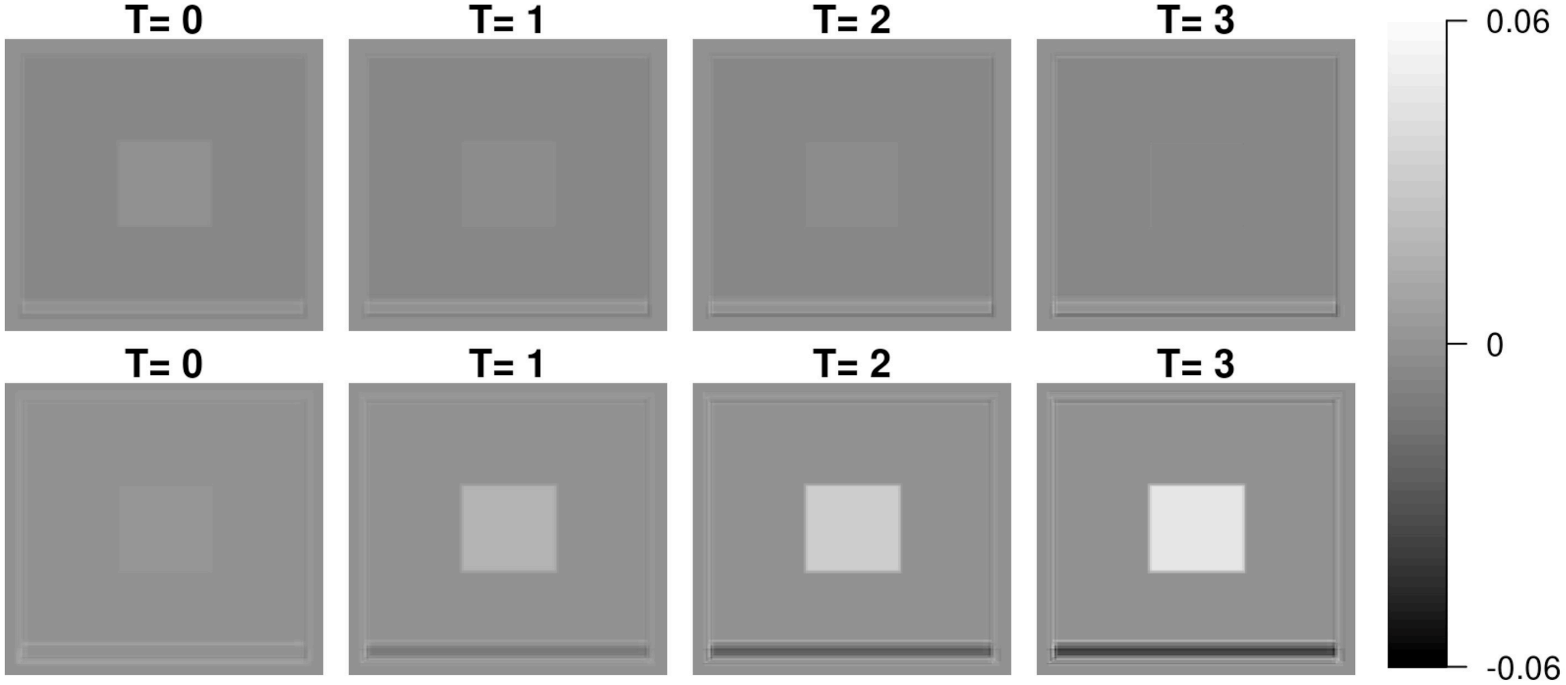
**First and fifth subject-specific components (Φ_***X***_) at time 0 (baseline), 1, 2, and 3**.

An advantage of LFPCA is its ability to couple baseline and longitudinal variation. The longitudinal component is added to the baseline with the time used as a multiplicative weight. Figure 4 illustrates the temporal trajectory of principal component loadings. We display only the first component, which does not appear to change over time. This pattern is replicated in the first four components. This indicates that the first four components mostly represent baseline variation. The fifth component loading does appear to change over time, while the baseline loading has relatively lower intensities compared to the longitudinal loading.

To summarize, our simulation studies convincingly demonstrate the power and flexibility of LFPCA to address some of key challenges of brain imaging. In particular, LFPCA managed to estimate and separate longitudinal and cross-sectional variation in a complex imaging simulation design with registration errors. The main part of the analysis can be automated and performed robustly with no operator input. We also applied a classical VBM-linear mixed effect model for the simulated data. As we expected, the linear mixed effect model could identify linear trend in the ventricular area (V), but it did not find significant trend in other areas (W and G) due to low longitudinal changes in signal and high visit-to-visit variation.

### 3.2. Classical VBM analysis using linear mixed model

In this section, we apply a standard VBM analysis to the MS cohort described in Section 2. This analysis focuses on the population mean of the longitudinal trend $\beta_{1}^{1}\left(v\right)$. After an FDR correction (Benjamini and Yekutieli, 2001) combined with cluster level thresholding, there are significant clusters with spatial extent more than 20 voxels. Table 1 shows information about the significant clusters, including cluster size, maximum or minimum *t*-values within each cluster and its location, and center of gravity of the clusters. We do not include an image of VBM results, since the clusters are very sparse having small cluster sizes. The results show that the longitudinal patterns do not appear to be significant for the most of brain regions. We suspect that it is because of the large variation within and between images due to real anatomic variation as well as registration error. We apply HD-LFPCA to uncover more subtle underlying variation.

**Table 1:** **Significant clusters of GM/WM/VN VBM results**.

		**Cluster size**	**t-MAX^a^ (or MIN)**	**t-MAX(or MIN)^b^**			**t-COG^c^**		
				***X***	***Y***	***Z***	***X***	***Y***	***Z***
GM	Atrophy	112	−6.34	139	86	67	139	90	68.9
		79	−6.36	118	158	35	116	158	35.0
		40	−5.84	122	166	41	121	166	41.0
		28	−6.13	136	148	80	136	149	79.8
		15	−5.36	143	167	89	143	166	89.3
VN	Enlargement	111	5.59	161	151	70	157	152	72.4
WM	Enlargement	154	6.30	118	150	76	117	150	75.6
		100	5.75	127	111	85	128	111	84.8
	Atrophy	210	−5.97	126	83	59	124	83.7	57.6
		157	−5.77	106	98	83	107	98	83.5
		31	−5.46	118	154	37	118	154	36.7

*All clusters are small (< 250) and spatially scatter*.

a *Maximum t-value for positive values and minimum t-value for negative values*.

b *Location of maximum (minimum) Z-value (Z-MAX(MIN))*.

c *Centre of gravity (COG) of the cluster*.

### 3.3. HD-LFPCA results

**Figure 5:**
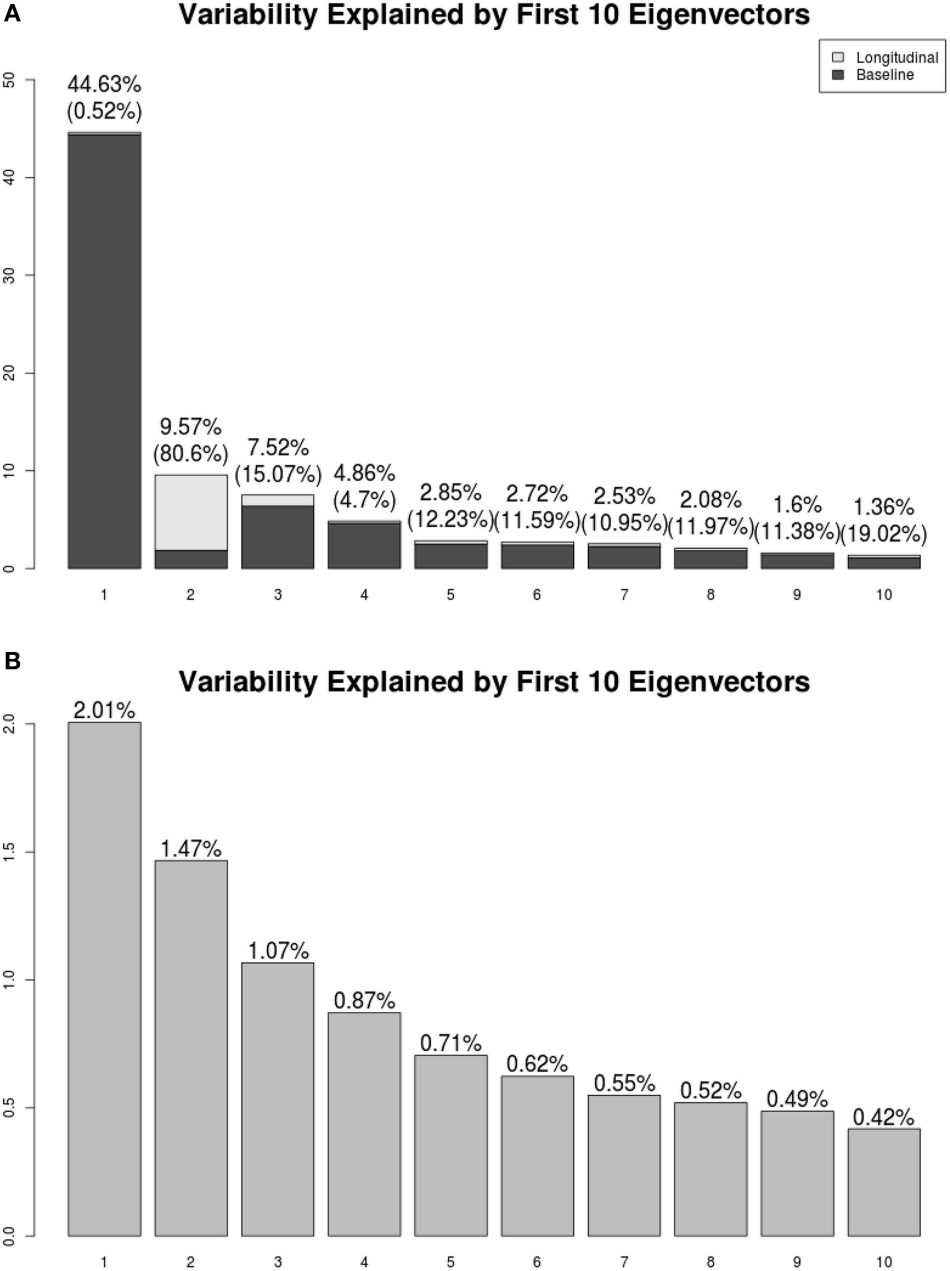
**Variation explained by (A) subject-specific components Φ_***X***_, (B) subject-visit-specific components Φ_***W***_**. Ratios of the longitudinal components to the LFPCs. The longitudinal components of the second LFPC explains 81% of the component, while those of other components have relatively low contributions.

We present the LFPCA results for ventricular RAVENS images in Figure 5. Figures 5A,B display the amount of variation explained by subject-specific components and subject and visit-specific components to the total variation, respectively. Figure 5A displays variation explained by the first 10 subject-specific components along the proportion of longitudinal variation represented within each subject-specific component. Each bar's height represents the percent of variation explained by each subject-specific component. It is color coded by the proportion of the variation explained by the longitudinal component and the baseline component. The top of each bar displays the variation explained by the subject-specific component; the fraction of that variation that is explained by the longitudinal component is given in parentheses.

The first subject-specific LFPC explains 45% of the overall variation, almost completely due to the cross-sectional part. The longitudinal part explains 81% of the variation within the second subject-specific LFPC. Figure 5B displays variation explained by the first 10 subject-visit-specific components to the total variation. The remaining LFPCs explain less than 1% of the total variation.

Most of the subject-specific LFPCs are driven by cross-sectional variation, which possibly include registration errors. The longitudinal changes are mainly captured by the second LFPC, which explains about 8% of the total variation. This provides an explanation as to why traditional VBM using linear mixed models did not find meaningful longitudinal patterns.

**Figure 6:**
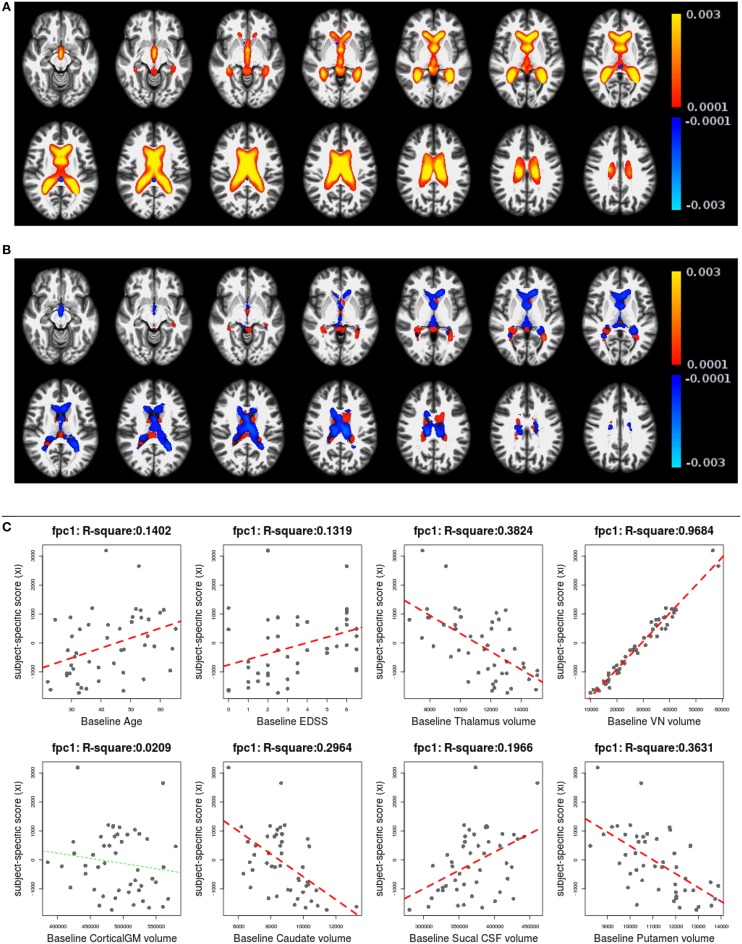
**The first subject-specific LFPC and scores**. **(A)** The baseline map, **(B)** the longitudinal map, **(C)** the first subject-specific LFPC scores and covariates of interest. The baseline map represents cross-sectional variation while the longitudinal map has lower loadings. The LFPC scores have higher correlations with baseline age, EDSS, volumes of GM substructures (thalamus, caudate, putamen), ventricle, and sulcal CSF. The correlation with ventricluar volume is very high, which confirms that the first LFPC represents cross-sectional ventricle size.

**Figure 7:**
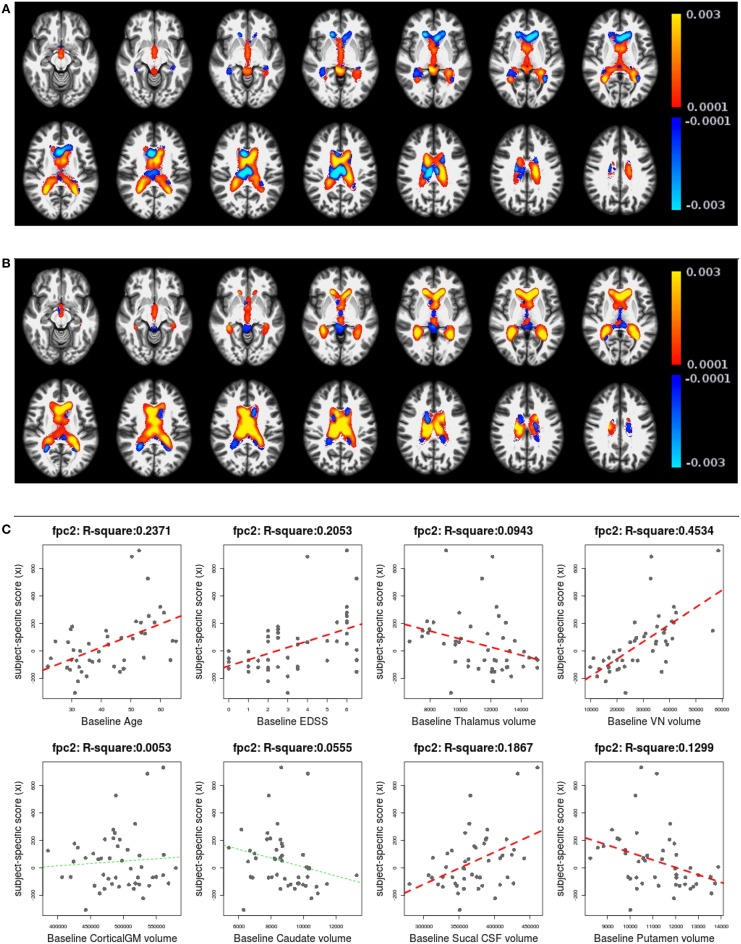
**The second subject-specific LFPC and scores**. **(A)** The baseline map, **(B)** the longitudinal map, **(C)** the second subject-specific LFPC scores and covariates of interest. The baseline map has relatively lower loadings while the longitudinal map shows an enlargement pattern in the ventricles. The LFPC scores have higher correlations with baseline age, EDSS, volumes of gray matter substructures (thalamus, putamen), ventricle, and sulcal CSF. The correlations with baseline age and EDSS scores are higher than those of the first LFPC scores.

Figures 6, 7 shows the first two pairs of LFPCs of ventricles. Figures 6A,B show the baseline and longitudinal components of the first LFPC. The LFPC loadings are color-coded with a red-yellow color scheme for positive values and blue-cyan for negative values. The first components reveal baseline ventricular morphometric variation, while the longitudinal component has relatively lower intensities. To investigate the characteristics of the first component, we fit the linear regression with covariates of interest and volumes of 6 ROIs obtained by the Lesion-TOADS segmentation algorithm. Figure 7C displays scatter plots of the LFPC scores with covariates, baseline age, baseline Expanded Disability Status Scale (EDSS) score, and volumes of 6 ROIs (thalamus, ventricle, cortical gray matter volume, caudate, sulcal CSF, putamen). The dashed lines overlaid on the scatter plots are the linear regression lines and are colored as red when the linear trend is significant and colored as green otherwise.

The significant correlation between the first subject-specific score and baseline VN volume (first row, fourth column) confirms that the first component represents baseline variation (*R*^2^: 0.9684), i.e., a subject with a positive score has larger ventricles at the baseline. The scores are significantly correlated to the subject's baseline age (*R*^2^: 0.1402) and three gray matter ROIs (thalamus, caudate, and putamen).

Figures 7A,B display the baseline and longitudinal components of the second subject-specific component, respectively. A subject with a positive score tends to have a larger regional volume at the baseline (yellow-red colored voxels in Figure 7A) and longitudinal enlargement. The second subject-specific scores have significant correlations with the baseline age, EDSS, thalamus volume, VN volumes, sulcal CSF volume and putamen volume. The second component scores have higher correlation with baseline age (*R*^2^: 0.2371) and EDSS (*R*^2^: 0.2053) than the first component scores that represent cross-sectional variation. This indicates that the spatial patterns of longitudinal enlargement in ventricles are superior for modeling disease progression and age compared to simple ventricular volume measures.

**Figure 8:**
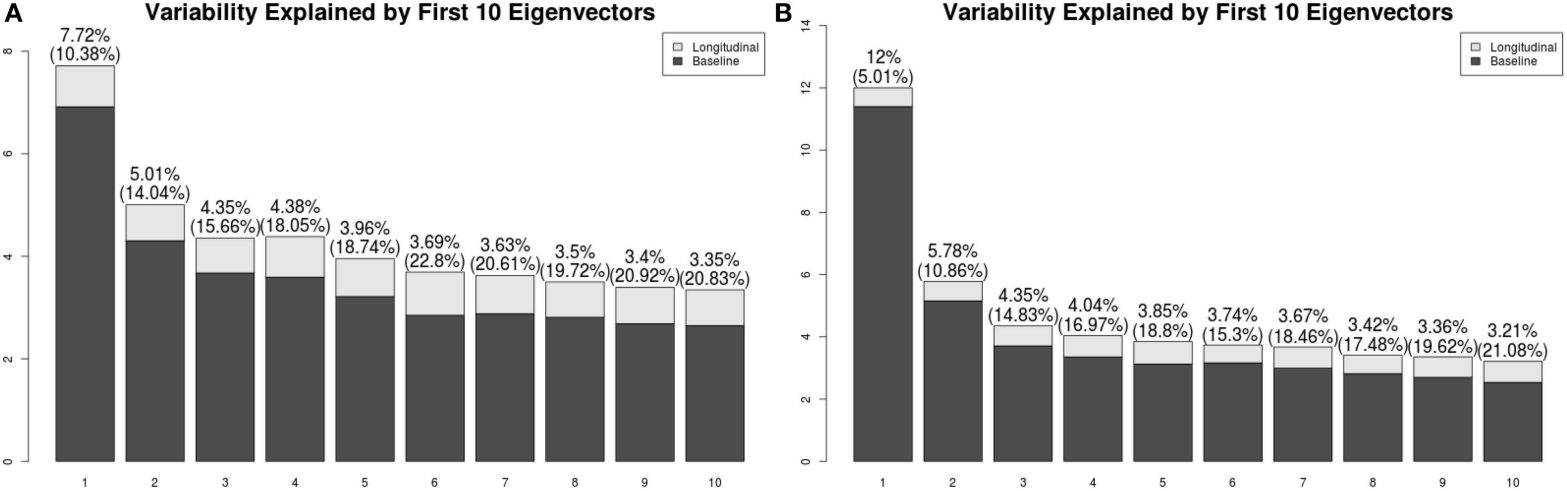
**Variation explained by first 10 subject-specific LFPCs in RAVENS maps**. **(A)** Gray matter, **(B)** white matter.

We have applied a similar analysis to gray matter and white matter RAVENS images. Figure 8 shows variation explained by first 10 subject-specific LFPCs in gray matter and white matter RAVENS images. In gray matter, around 20% variation comes from the longitudinal part across all subject-specific LFPCs. Lower proportions of variation, around 15%, are explained by the longitudinal part in white matter. Unlike the ventricles, any subject-specific component of gray and white matter is not dominated by the longitudinal part. We speculate it is due to spatial heterogeneity of longitudinal brain atrophy, which may depend on subject-specific disease progression. In the correlation analyses with age and EDSS scores, the first LFPC component of the gray matter was significantly associated with age (*r* = −0.48, *p* < 0.001) and EDSS (*r* = −0.57, *p* < 0.001) indicating gray matter thinning is highly associated with age and MS progression, while other components were not significantly associated with age or EDSS. For white matter, LFPC1 was not significantly associated with age (*r* = −0.07, *p* = 0.63) but with EDSS (*r* = −0.32, *p* = 0.03). LFPC2 was significantly associated with both age (*r* = −0.36, *p* = 0.01) and EDSS (*r* = −0.34, *p* = 0.02). Although those LFPC components did not contain substantial longitudinal changes, the results indicate that local atrophy in the white matter can inform disease progression.

As described above, LFPCA is a useful dimension reduction tool for high-dimenstional longitudinal data. In this section, we illustrated how the LFPC scores an be used in the correlation analyses. Further, LFPCA scores can be used as predictors or outcomes in regression analyses, classification or cluster analysis.

## 4. Discussion

In this manuscript, we described and evaluated a coherent methodology for the study of longitudinal RAVENS—or other methodological—volumetric imaging studies. Our simulation studies demonstrate that LFPCA tightly links the analysis methodology with the morphometric image processing stream. We demonstrated that LFPCA can uncover interesting, yet subtle, directions of longitudinal variation in a case where independent voxel-level investigations fail. Of note, this study represents the first application of the high dimensional variation of LFPCA to voxel-based morphometric analysis. Related work includes Zipunnikov et al. (2014), who investigated DTI imaging data and Zipunnikov et al. (2011a) and Zipunnikov et al. (2011b), who studied RAVENS images with cross-sectional and clustered (but not longitudinal) settings. Moreover, this manuscript represents the first application of LFPCA to morphometric imaging in multiple sclerosis.

A key insight from the simulation studies is the ability of LFPCA to uncover interesting directions of variation in the presence of errors from registration to a template. Previously, registration errors were handled via either extremely aggressive smoothing during post-registration processing or by improved normalization algorithms. While improved algorithms are certainly a desirable goal, all normalization algorithms must be tuned and suffer from trade offs (such as bias and variance). Our results suggest the possibility of employing less aggressive normalization.

The performance of LFPCA depends on the number of subjects, the number of time points, and time span over which data is collected. In designing imaging studies for LFPCA, having both a large number of subjects and a large number of visits may be challenging to obtain. Simulation studies we have conducted during the process of examining LFPCA showed that LFPCA performed well as long as we have either many subjects with smaller number of visits or smaller number of subjects with many visits. It is recommended to make the time span over which data is collected roughly similar across subjects, and long enough to observe longitudinal changes.

In our study of MS, we found that the majority of variation is focused in cross-sectional components. This will likely be true in any study of adults, as variation in head size, brain size and intracranial volume will vary far more substantially than longitudinal decline, not unlike if one were to study adult cross-sectional and longitudinal trends in heights. It would be of interest to apply LFPCA to developmental populations or populations with severe progressive brain disorders significantly after disease onset.

The correlation between subject-specific LFPC scores of ventricles and EDSS indicates that EDSS is better associated with longitudinal ventricular enlargement than baseline ventricular size. This implies ventricular enlargement is a sensitive measurement of disease progression. Some cross-sectional MS patient studies have reported that brain atrophy is related to irreversible clinical disability in (MS) and ventricular enlargement may be a sensitive marker of this tissue loss that is seen at all stages of MS (Turner et al., 2001; Benedict et al., 2005; Hildebrandt et al., 2006; Tekok-Kilic et al., 2007). In existing longitudinal studies, longitudinal ventricular enlargement and gray matter atrophy have been detected in both ROI and VBM with paired *t*-test or factor models (Simon et al., 1999; Dalton et al., 2002, 2006; Kalkers et al., 2002; Sepulcre et al., 2006; Lukas et al., 2010; Bendfeldt et al., 2009), which agrees with our finding. Unlike other methods, LFPCA is able to show spatially heterogeneous patterns of longitudinal enlargement, which ROI based methods cannot provide.

In the manuscript, we employed a registration strategy similar to Ashburner and Ridgway (2012). Recent developments in longitudinal registration algorithms (e.g., 4DHammer: Shen et al., 2003, CLASSIC: Shen et al., 2005, GLIRT: Wu et al., 2010, Quarc: Holland et al., 2011) are potentially capable of providing higher accuracy in tracking within subject anatomical changes. Improvements in registration would likely increase the sensitivity of LFPCA to subject-specific signals. However, visit-to-visit variation caused by image acquisition inconsistencies or large anatomical differences between subjects often cannot be corrected by registration. An advantage of the proposed method is that it can simultaneously quantify and characterize both cross-sectional and longitudinal signals of interest in the presence of potentially large amounts of visit-to-visit variation.

As demonstrated previously for longitudinal diffusion imaging analysis, and here for longitudinal voxel-based morphometry, LFPCA is a compelling alternative to linear mixed model analysis for exploring spatial patterns of anatomical variation within and across subjects. We emphasize that this approach is not limited to a specific brain modality. Besides neuroimaging, we look forward to seeing this method is applied to many other exciting studies including epigenetics. For example, genome-wide DNA methylation data collected at multiple time points could be analyzed to study mechanisms of epigenetic changes related to certain diseases (Martino et al., 2014) or environmental exposure (Martino et al., 2013). Another potential domain of application is for analyzing dynamic imaging data, such as functional MRI or motion imaging. Such data often possess much larger numbers of time points, which would be needed to model the more complex variations in signal.

The LFPCA method described here is designed to model a linear trajectory over time. Given a relatively small number of visits (e.g., three visits on average) it is not feasible to model non-linear trends. However, if the data are collected over greater than 5 time points, the modeling of non-linear trajectories is possible. Currently, we are under a preliminary development of a method to extend LFPCA for non-linear trends modeled using spline functions. Further investigation on the numerical stability and performance will be conducted in the near future.

## Funding

Research reported in this work was supported by National Institute of Health under award numbers R01NS070906, Z01NS003119, K01AG051348, R01HL12407 and R01NS060910. Support for this work included funding from the Department of Defense in the Center for Neuroscience and Regenerative Medicine.

### Conflict of interest statement

The authors declare that the research was conducted in the absence of any commercial or financial relationships that could be construed as a potential conflict of interest.
